# Antiproliferative effect of immunoliposomes containing 5-fluorodeoxyuridine-dipalmitate on colon cancer cells

**DOI:** 10.1038/sj.bjc.6690588

**Published:** 1999-08

**Authors:** G A Koning, A Gorter, G L Scherphof, J A A M Kamps

**Affiliations:** Department of Physiological Chemistry, Faculty of Medical Sciences, Groningen Institute for Drug Studies (GIDS), A Deusinglaan 1, AV Groningen, 9713, The Netherlands; Department of Pathology, Leiden University Medical Center, PO Box 9600, RC Leiden, 2300, The Netherlands

**Keywords:** immunoliposomes, monoclonal antibody, colon cancer, tumour targeting, 5-fluorodeoxyuridine, drug delivery

## Abstract

We have investigated the antiproliferative action towards CC531 colon adenocarcinoma cells of target cell-specific immunoliposomes containing the amphiphilic dipalmitoyl derivative of 5-fluorodeoxyuridine (FUdR-dP). FUdR-dP incorporated in immunoliposomes caused a 13-fold stronger inhibition of CC531 cell growth in vitro, during a 72-h treatment, than FUdR-dP in liposomes without antibody, demonstrating that the prodrug is efficiently hydrolysed to yield the active drug, FUdR, intracellularly. The intracellular release of active FUdR was confirmed by determining the fate of ^3^H-labelled immunoliposomal FUdR-dP. Treatments shorter than 72 h with FUdR-dP in immunoliposomes resulted in anti-tumour activities comparable to, or even higher than, that of free FUdR. The shorter treatments reflect more closely the in vivo situation and illustrate the potential advantage of the use of immunoliposomes over non-targeted liposomal FUdR-dP or free FUdR. Association of tumour cell-specific immunoliposomes with CC531 cells was up to tenfold higher than that of liposomes without antibody or with irrelevant IgG coupled, demonstrating a specific interaction between liposomes and target cells which causes an efficient intracellular delivery of the drug. Since biochemical evidence indicates a lack of internalization or degradation of the liposomes as such, we postulate that entry of the drug most likely involves the direct transfer of the prodrug from the immunoliposome to the cell membrane during its antigen-specific interaction with the cells, followed by hydrolysis of FUdR-dP leading to relatively high intracellular FUdR-levels. In conclusion, we describe a targeted liposomal formulation for the anticancer drug FUdR, which is able to deliver the active drug to colon carcinoma cells with high efficiency, without the need for the cells to internalize the liposomes as such. © 1999 Cancer Research Campaign


British Journal of Cancer (1999) **80**(11), 1718–1725
© 1999 Cancer Research Campaign
Article no. bjoc.1999.0588

### Antiproliferative effect of immunoliposomes containing 5-fluorodeoxyuridine-dipalmitate on colon cancer cells

**GA Koning[1]*, A Gorter[2], GL Scherphof[1] and JAAM Kamps[1]**

[1]Groningen Institute for Drug Studies (GIDS), Department of Physiological Chemistry, Faculty of Medical Sciences, University of Groningen, A Deusinglaan 1, 9713 AV Groningen, The Netherlands; [2]Department of Pathology, Leiden University Medical Center, PO Box 9600, 2300 RC Leiden, The Netherlands

**Summary** We have investigated the antiproliferative action towards CC531 colon adenocarcinoma cells of target cell-specific immunoliposomes containing the amphiphilic dipalmitoyl derivative of 5-fluorodeoxyuridine (FUdR-dP). FUdR-dP incorporated in immunoliposomes caused a 13-fold stronger inhibition of CC531 cell growth in vitro, during a 72-h treatment, than FUdR-dP in liposomes without antibody, demonstrating that the prodrug is efficiently hydrolysed to yield the active drug, FUdR, intracellularly. The intracellular release of active FUdR was confirmed by determining the fate of $^3$H-labelled immunoliposomal FUdR-dP. Treatments shorter than 72 h with FUdR-dP in immunoliposomes resulted in anti-tumour activities comparable to, or even higher than, that of free FUdR. The shorter treatments reflect more closely the in vivo situation and illustrate the potential advantage of the use of immunoliposomes over non-targeted liposomal FUdR-dP or free FUdR. Association of tumour cell-specific immunoliposomes with CC531 cells was up to tenfold higher than that of liposomes without antibody or with irrelevant IgG coupled, demonstrating a specific interaction between liposomes and target cells which causes an efficient intracellular delivery of the drug. Since biochemical evidence indicates a lack of internalization or degradation of the liposomes as such, we postulate that entry of the drug most likely involves the direct transfer of the prodrug from the immunoliposome to the cell membrane during its antigen-specific interaction with the cells, followed by hydrolysis of FUdR-dP leading to relatively high intracellular FUdR-levels. In conclusion, we describe a targeted liposomal formulation for the anticancer drug FUdR, which is able to deliver the active drug to colon carcinoma cells with high efficiency, without the need for the cells to internalize the liposomes as such.

**Keywords:** immunoliposomes; monoclonal antibody; colon cancer; tumour targeting; 5-fluorodeoxyuridine; drug delivery

Primary tumours of the gastrointestinal tract usually can be removed efficiently by surgery, but 50–60% of all patients develop metastases within the next 5 years, mostly in the liver (Vahrmeijer et al, 1995; Kemeny, 1996). Hepatic metastases are difficult to treat with current treatment regimens. Chemotherapy using fluoropyrimidines, often in combination with modulators like leucovorin, methotrexate or interferon, is most often used in these patients, but with a minimal increase in survival rates (Meropol et al, 1995). Hepatic arterial infusion of chemotherapeutics results in higher response rates and survival in preclinical and clinical studies (Marinelli et al, 1991; Kemeny, 1995), indicating that local drug application may offer good prospects.

5-Fluoro-2′-deoxyuridine (FUdR), a cytotoxic drug of the group of fluoropyrimidines, and its metabolite 5-fluorouridine (5-FU) are among the most often used cytotoxic drugs in the treatment of disseminated colorectal cancer. FUdR needs to be converted to FdUMP (5-fluoro-2′-deoxyuridine-5′-monophosphate) which is a potent inhibitor of thymidylate synthase, responsible for the generation of dTMP (2′-deoxy-thymidine-5′-monophosphate). The resulting lack of dTMP leads to inhibition of DNA-synthesis (Danenberg, 1977). FdUMP can be converted to FdUTP (FUdR-5′-triphosphate) which can be incorporated into DNA. FUdR can also be converted to 5-FU which is metabolized into FUTP (5-fluorouridine-5′-triphosphate), a substrate for RNA polymerase and is incorporated into RNA. Incorporation of FdUTP or FUTP into DNA or RNA, respectively, causes damage to these nucleic acids (Myers, 1981).

Liposomes have been shown to be potentially attractive carriers for anticancer drugs (Gregoriadis, 1995; Allen, 1997; Storm et al, 1997) because they prevent the drug from degradation in circulation and may diminish toxic side-effects by altering pharmacokinetic behaviour. Besides, it has been shown that liposomes, and consequently the liposomal drug, may accumulate in tumour areas provided that the liposomes are small and have a sufficiently long circulation time (Papahadjopoulos et al, 1991; Vaage et al, 1997). The latter can be achieved for example by the ganglioside $GM_1$ or polyethyleneglycol (PEG) (Allen et al, 1987, 1991). A further increase in tumour localization of liposomes may be achieved by the coupling of a tumour cell-specific monoclonal antibody to the liposomes (immunoliposome), to allow specific interaction with the tumour cells (Martin et al, 1982; Maruyama et al, 1990; Ahmad et al, 1993).

In this study we investigated the possibility of using liposomes to deliver FUdR into rat CC531 colon adenocarcinoma cells. A liver metastases model of this tumour has been developed, offering a representative model for liver metastases of colon cancer in humans (Marquet et al, 1984; Thomas et al, 1993; Dingemans

*Received 16 September 1998*
*Revised 19 January 1999*
*Accepted 16 February 1999*

*Correspondence to*: GL Scherphof

*Present address: Department of Pharmaceutics, University of Utrecht, PO Box 80082, 3508 TB Utrecht, The Netherlands et al, 1994). The monoclonal antibody, CC52, specific for a surface antigen on CC531 tumour cells (Beun et al, 1992, 1993), was used as a specific targeting device.

Since FUdR is not efficiently retained in liposomes under in vivo conditions we synthesized a lipophilic dipalmitoyl ester of FUdR, FUdR-dP (Nishizawa et al, 1965), to anchor the drug in the liposomal bilayer (Schwendener et al, 1985; Van Borssum Waalkes et al, 1993). We showed that FUdR-dP remains firmly associated with the liposomes and does not exchange with serum components such as albumin or lipoproteins or with liposomal bilayers (Van Borssum Waalkes et al, 1993).

FUdR-dP was incorporated in several types of (immuno)liposomes varying in size and composition. All formulations were tested for their antiproliferative activity towards CC531 colon cancer cells in vitro. To study the interaction of the liposomes with CC531 cells and the possible mechanism of delivery of the drug to the cells, we assessed association, internalization and degradation of the liposomes and of the lipophilic prodrug.

### MATERIALS AND METHODS

### Materials

FUdR, *N*-succinimidyl-*S*-acetylthioacetate (SATA) and cholesterol (Chol) were obtained from Sigma (St Louis, MO, USA). Egg yolk phosphatidylcholine (PC), maleimido-4-(*p*-phenylbutyryl)-phosphatidylethanolamine (MPB-PE) and methoxypoly(ethyleneglycol)$_{2000}$-distearoylphosphatidylethanolamine ($PEG_{2000}$-DSPE) were purchased from Avanti Polar Lipids (Alabaster, AL, USA). Bovine brain ganglioside $GM_1$ was obtained from Calbiochem (La Jolla, CA, USA) cholesteryl-[$^{14}$C]-oleate, [$^3$H]-cholesteryloleylether and [methyl-$^3$H]-thymidine were obtained from Amersham (Buckinghamshire, UK), 5-[6-$^3$H]-fluoro-2′-deoxyuridine ($^3$H-FUdR) was obtained from DuPont NEN (Wilmington, DE, USA). All other chemicals were analytical grade or the best grade available. Polycarbonate filters for liposome extrusion using a high-pressure extruder were from Costar (Cambridge, MA, USA). Filters for liposome extrusion using the Liposofast Basic extruder were from Avestin Inc. (Ottawa, ON, Canada).

### Monoclonal antibody

The monoclonal antibody CC52 ($IgG_1$), recognizing a surface antigen on CC531 colon adenocarcinoma cells, was developed in the department of Pathology, Leiden University Medical Center, The Netherlands (Beun et al, 1992). A murine $IgG_1$ monoclonal antibody against human B-cells used as an irrelevant control antibody, was a gift from Dr C Thomas, Department of Physiological Chemistry, Groningen. Both antibodies were purified from culture supernatant by protein A-sepharose (Pharmacia, Woerden, The Netherlands) chromatography, according to the manufacturer's instructions.

### Synthesis of FUdR-dP

FUdR-dP was synthesized as described earlier (Van Borssum Waalkes et al, 1993). Briefly, 0.4 mmol of FUdR was dissolved in 1 ml of dimethylacetamide to which 0.8 mmol of palmitoylchloride was added. For the synthesis of $^3$H-FUdR-dP, 25 µCi of $^3$H-FUdR, labelled in the uracil moiety, was added per µmol of FUdR. The mixture was incubated overnight under constant shaking at 40°C. To the mixture distilled water was added, the resulting white precipitate was applied to a glass filter and washed with distilled water. FUdR-dP was recrystallized three times from methanol. Purity of the compound was checked by thin-layer chromatography.

### Liposomes

Liposomes were composed of PC and 30 or 40 mol% Chol. Liposomes to which CC52 antibodies were coupled contained 0.025 mol MPB-PE per mol lipid (cholesterol and phospholipid). Long-circulating liposomes were prepared by incorporating 0.06 mol $GM_1$ or 0.04 mol $PEG_{2000}$-DSPE per mol lipid. FUdR-dP and $^3$H-FUdR-dP (specific activity 25 µCi $\mu mol^{-1}$) was incorporated in the liposomes at a level of 2 or 3 mol %. When required, liposomes were labelled with trace amounts of [$^3$H]-cholesteryl-oleylether (1 Ci $mol^{-1}$ lipid) and cholesteryl-[$^{14}$C]-oleate (0.4 Ci $mol^{-1}$ lipid). Lipids dissolved in chloroform:methanol (9:1), were mixed and dried under nitrogen pressure, dissolved in cyclohexane and lyophilized. Lipids were hydrated in HN-buffer (10 mM *N*-2-hydroxyethylpiperazine-*N*'-2-ethanesulphonic acid (HEPES), 135 mM sodium chloride), pH 7.4 or, for coupling of antibodies in HN-buffer, pH 6.7. Liposomes were sized by repeated extrusion through filters with a pore size of 200 or 50 nm using a high-pressure extruder (Lipex, Vancouver, Canada) or a LiposoFast Basic (Avestin Inc. Ottawa, Canada). Phospholipid phosphorus of each liposome preparation was determined by a phosphate assay after perchloric acid destruction (Böttcher et al, 1961). Total liposomal lipid concentrations were calculated, taking into account the amount of cholesterol in the liposome preparations. Particle size and size distribution were determined by dynamic laser light scattering with a Nicomp model 370 submicron particle analyser (Nicomp, Santa Barbara, CA, USA), using the volume distribution curves for obtaining the diameter of the liposomes. Liposomes extruded through 200 nm had a diameter of 179 nm ± 61 nm (mean ± standard deviation (s.d.)) and are referred to as 200-nm liposomes; liposomes extruded through 50 nm had a diameter of 82 nm ± 25 nm and are referred to as 50-nm liposomes.

### Coupling of antibodies to liposomes

Monoclonal antibodies were coupled to MPB-PE containing liposomes by a sulphhydryl-maleimide coupling method as described previously (Derksen et al, 1985). Briefly, free sulfhydryl groups were introduced in the antibody, using the heterobifunctional reagent SATA. Free SATA was separated from the derivatized antibody by gel permeation chromatography using Sephadex G-50. Antibody with reactive sulfhydryl groups, induced by deacetylating the ATA-protein, were incubated with MPB-containing liposomes for 4 h at room temperature at a ratio of 0.3 mg antibody per µmol of lipid. *N*-ethylmaleimide (8 mM in HN-buffer, pH 7.4) was added to cap unreacted sulfhydryl groups. Unconjugated antibody was separated from liposomes by flotation on a metrizamide gradient as described before (Kamps et al, 1996). Hereafter, immunoliposomes were extensively dialysed against HN-buffer, pH 7.4 and characterized by determining protein content using IgG as a standard (Petterson, 1977), phospholipid phosphorus content and particle size. PC/Chol-immunoliposomes contained 67–69 µg, PC/Chol-$GM_1$-immunoliposomes 52–71 µg and PC/Chol-PEG-immunoliposomes 17–38 µg CC52 per µmol of liposomal lipid. PC/Chol-PEG-liposomes contained 92 µg $\mu mol^{-1}$ of irrelevant

© 1999 Cancer Research Campaign 

murine $IgG_1$. Liposomes increased in size as a result of the coupling of CC52; 200-nm liposomes increased by 15%, 50-nm PC/CH-liposomes by 80%, 50-nm $GM_1$-liposomes by 39% and CC52-PC/Chol/PEG-liposomes by 7%, no aggregates were observed. Liposomes were stored at 4°C under nitrogen and used within 2 weeks after preparation.

### Cell culture

CC531 colon adenocarcinoma is a 1,2-dimethylhydrazine-induced carcinoma of the colon of WAG/Rij-rats (Marquet et al, 1984). Cells were maintained in 75-$cm^2$ culture flasks (Costar, Cambridge, MA, USA) in RPMI-1640 medium with 25 mM HEPES (Gibco BRL, Breda, The Netherlands) supplemented with 10% heat-inactivated fetal calf serum (FCS) (Gibco), fresh L-glutamine (2 mM) and penicillin–streptomycin (100 U $ml^{-1}$ and 100 μg $ml^{-1}$ respectively) (Gibco) at 37°C in a humidified atmosphere consisting of 5% carbon dioxide in air. Cells were subcultured at 80% confluency.

### In vitro proliferation assay

After trypsinization, CC531 cells were plated in 96-well plates (Costar) in culture medium, at a density which allows cells to be in the log-phase of growth during determination of the proliferation; 2000 cells per well for 72-h treatment and 3500 cells per well for 48-h treatment. Cells were allowed to adhere overnight, whereafter varying amounts of the different test compounds were added. In incubations with liposomes not containing FUdR-dP, the same amounts of liposomal lipid were added as for FUdR-dP containing liposomes. Twenty hours before terminating the incubations, 0.06 μCi of [methyl-$^3$H]-thymidine (specific activity 5 Ci $mmol^{-1}$) was added per well. At the end of the incubations, cells were washed twice with phosphate-buffered saline (PBS) (137.9 mM sodium chloride, 2.7 mM potassium chloride, 8.1 mM sodium phosphate and 1.5 mM $KH_2PO_4$, pH 7.4) and lysed in sample buffer (0.125 M tri-sodium-citrate, 4% sodium dodecyl sulphate (SDS), 0.1 M β-mercaptoethanol and 0.2 M glycerol). Samples were harvested for liquid scintillation counting with Ultima Gold XR (Packard Instruments, Groningen, The Netherlands). Proliferation was expressed as the percentage $^3$H-thymidine incorporation of the treated cells compared with untreated cells. The drug concentration causing 50% growth inhibition ($IC_{50}$) was calculated by interpolation of the two values closest to 50% inhibition on the growth inhibition curve.

### Interaction of (immuno)liposomes and incorporated $^3$H-FUdR-dP with CC531 cells

After trypsinization CC531 cells were plated on 6- or 24-well plates (Costar) and left to grow for 2 days. One hour before the start of the experiment cells were washed with PBS and fresh medium with or without 10% FCS was added to the cells. For determining association of liposomes, CC531 cells were incubated in 24-well plates for 3 h in the absence of FCS with 200 nmol $ml^{-1}$ tumour cell-specific immunoliposomes (CC52-PC/Chol/PEG-liposomes), control liposomes without antibody (PEG-liposomes) or liposomes with irrelevant murine $IgG_1$ coupled ($IgG_1$-PC/Chol/PEG-liposomes), all labelled with $^3$H-COE. Subsequently, medium was removed, cells were thoroughly washed with ice-cold PBS and lysed using 0.4 M sodium hydroxide. Cell-associated radioactivity was determined and normalized for the amount of cellular protein as determined by protein assay (Lowry et al, 1951) using BSA (bovine serum albumin) as a standard. For determining liposome uptake and degradation, cells were incubated in 6-well plates for 4, 8, 24 or 48 h with 100 nmol CC52-PC/Chol-liposomes double-labelled with $^3$H-cholesteryloleylether ($^3$H-COE), a non-degradable marker and cholesteryl-[$^{14}$C]-oleate ($^{14}$C-COA), an intracellularly degradable marker which results in release of $^{14}$C-oleic acid from the cells as described before (Derksen et al, 1988). Incubations were stopped by removing the liposome-containing medium followed by thorough washing of the cells with ice-cold PBS. Cells were harvested by trypsinization and centrifugation. Cell-associated labels were determined by combining the radioactivity in the supernatant and in the cell pellet after lysing the cells with SDS and were normalized for the amount of cellular protein as determined by protein assay. The $^3$H:$^{14}$C ratio of the cell-associated radioactivity was calculated and taken as a measure of the degradation of the $^{14}$C-COA and thus of liposome degradation.

For determining the uptake of immunoliposome-incorporated $^3$H-FUdR-dP, CC531 cells were incubated in the absence of FCS in 6-well plates for 3 h with 100 nmol $^3$H-FUdR-dP containing CC52-MPB-liposomes, followed by a liposome-free incubation period of 6 h. Radioactivity was measured in the medium, in the supernatant after trypsin treatment with 0.05% trypsin for 10 min and centrifugation (cell-bound label), and in the trypsin-treated cell pellet (intracellular label) after lysing the cells with 10% SDS. To discriminate between hydrophobic FUdR-dP and hydrophilic FUdR or metabolites (hydrolysed prodrug), chloroform:methanol extractions were performed by mixing sample, methanol, chloroform and PBS in a ratio of 4:10:9:5 (vol/vol). The chloroform and methanol:water layers were isolated and $^3$H-label was measured by liquid scintillation counting and normalized for the amount of cellular protein.

### Statistical evaluation

Statistical significance of differences was evaluated by a two-tailed unpaired Student's *t*-test.

### RESULTS

### Increased anti-tumour activity of FUdR-dP in immunoliposomes

In Figure 1 the antiproliferative effects on CC531 cells of a 72-h treatment with FUdR-dP incorporated in 200-nm $GM_1$-containing immunoliposomes are compared with those of 200-nm $GM_1$-liposomes without antibody and free FUdR. Liposomes not containing FUdR-dP had no effect on tumour cell proliferation (not shown). FUdR-dP in 200-nm CC52-$GM_1$-liposomes was much more effective in inhibiting tumour cell proliferation than FUdR-dP in $GM_1$-liposomes without the CC52 antibody.

In Table 1 the FUdR-concentrations resulting in a 50% inhibition of tumour cell proliferation ($IC_{50}$) are given to enable direct comparison of the effect of various liposomal formulations and treatment conditions on tumour cell toxicity. In case of immunoliposomal FUdR-dP the 50% inhibition of proliferation was reached at a 13-fold lower drug-concentration than with FUdR-dP in liposomes without antibody. At FUdR-concentrations of $10^{-6}$ M and higher, FUdR-dP-containing immunoliposomes were able to

 © 1999 Cancer Research Campaign

**Table 1** $IC_{50}$ values of inhibition of CC531 colon cancer cell proliferation by liposomal FUdR-dP formulations and FUdR

| Liposome composition | Chol (mol%) | Size (nm) | Treatment (h) | $IC_{50}$ FUdR (M) | $IC_{50}$ FUdR (ng $ml^{-1}$) |
|---|---|---|---|---|---|
| PC/Chol/FUdR-dP | 30 | 200 | 72 | $3.1\times10^{-7}$ | 77 |
| PC/Chol/$GM_1$/FUdR-dP | 30 | 200 | 72 | $6.9\times10^{-7}$ | 170 |
| CC52-PC/Chol/$GM_1$/FUdR-dP | 30 | 200 | 72 | $5.5\times10^{-8}$ | 13 |
| CC52-PC/Chol/FUdR-dP | 30 | 50 | 72 | $1.2\times10^{-8}$ | 3 |
| CC52-PC/Chol/$GM_1$/FUdR-dP | 30 | 50 | 72 | $3.4\times10^{-8}$ | 8 |
| CC52-PC/Chol/$GM_1$/FUdR-dP | 40 | 50 | 48 | $1.2\times10^{-5}$ | 2954 |
| CC52-PC/Chol/PEG/FUdR-dP | 40 | 50 | 48 | $1.1\times10^{-5}$ | 2708 |
| CC52-PC/Chol/$GM_1$/FUdR-dP | 40 | 50 | 72 | $2.2\times10^{-8}$ | 5 |
| CC52-PC/Chol/PEG/FUdR-dP | 40 | 50 | 72 | $1.0\times10^{-7}$ | 24 |
| Free FUdR | | | 72 | $2.1\times10^{-9}$ | 1 |
| CC52-PC/Chol/$GM_1$/FUdR-dP | 40 | 50 | 24 + 48[a] | $3.7\times10^{-8}$ | 9 |
| CC52-PC/Chol/PEG/FUdR-dP | 40 | 50 | 24 + 48[a] | $5.0\times10^{-6}$ | 1234 |
| Free FUdR | | | 24 + 48[a] | $8.6\times10^{-7}$ | 213 |

[a]Cells were treated for 24 h, followed by 48 h incubation in medium without liposomal or free drug.

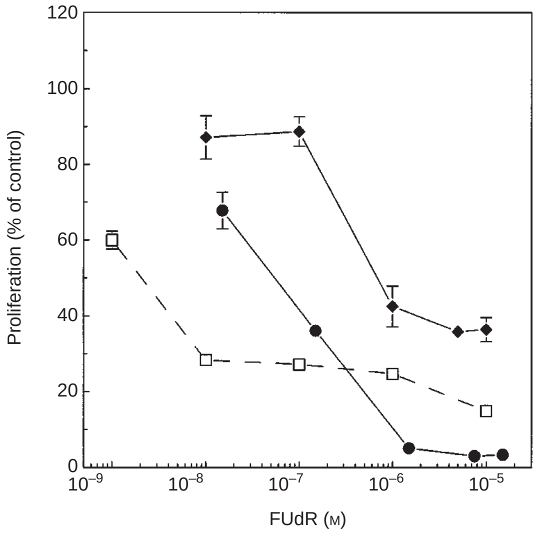

**Figure 1** Proliferation of CC531 tumour cells as a function of the amount of FUdR added as FUdR-dP in 200-nm liposomes or free FUdR. Proliferation was assayed by [3]H-thymidine incorporation and expressed as percentage of label incorporated in untreated cells (± s.e.m.). Cells were incubated for 72 h with: PC/Chol/$GM_1$/FUdR-dP liposomes (◆) ($n$ = 6), CC52-PC/Chol/$GM_1$/FUdR-dP-liposomes (●) ($n$ = 6) or free FUdR (□) ($n \geq 12$)

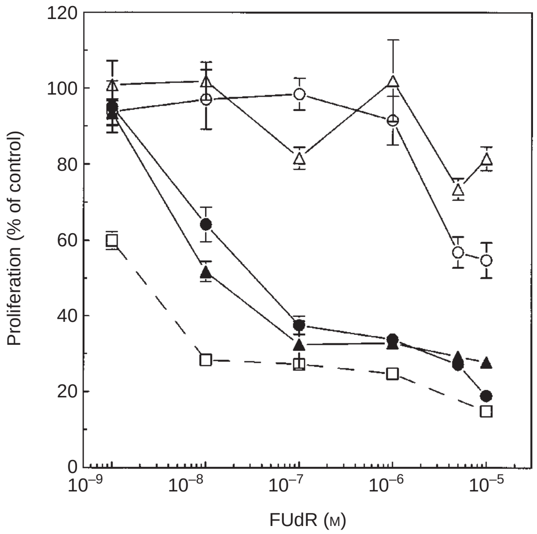

**Figure 2** Proliferation of CC531 tumour cells as a function of the amount of FUdR added as FUdR-dP in 50-nm liposomes (30 mol % Chol) or free FUdR. Cells were incubated for 72 h with CC52-liposomes composed of: PC/Chol (Δ), PC/Chol/$GM_1$ (○), PC/Chol/FUdR-dP (▲) or PC/Chol/$GM_1$/FUdR-dP (●) (all $n$ = 6) or with free FUdR (□) ($n \geq 12$). Proliferation is expressed as percentage of the proliferation of untreated cells (average ± s.e.m.)

inhibit tumour cell growth almost completely, in contrast to FUdR-dP in $GM_1$-liposomes without CC52, which caused a maximum inhibition of 60%, while free FUdR inhibited cell growth maximally by 85%. At the lower concentration range free FUdR was more effective in inhibiting CC531 proliferation.

### Antiproliferative activity of FUdR-dP in 50-nm immunoliposomes

In vivo, target site accessibility will be favourably influenced by decreasing liposome size and increasing circulation time by incorporating substances like $GM_1$ or PEG.

Antiproliferative effects of FUdR-dP in 50-nm CC52-liposomes are shown in Figure 2. At FUdR-concentrations of $10^{-7}$ M and higher, FUdR-dP incorporated in 50-nm CC52-liposomes inhibited tumour cell growth to the same extent as free FUdR, whereas at lower concentrations free FUdR is more effective than immunoliposomal FUdR-dP. CC52-liposomes without FUdR-dP had only minor effects on CC531 proliferation. Up to FUdR-concentrations of $10^{-7}$ M no significant difference in antiproliferative potency was observed between FUdR-dP containing 50-nm (Figure 2) or 200-nm (Figure 1) CC52-$GM_1$-liposomes. Both liposome preparations showed comparable $IC_{50}$-values (Table 1). However, a notable difference between the smaller and the larger liposomes in the extent of growth inhibition was observed at concentrations higher than $10^{-7}$ M. With the 50-nm CC52-liposomes a proliferation inhibition of maximally 80%, in contrast to the 100% for the 200-nm CC52-liposomes.

*© 1999 Cancer Research Campaign*

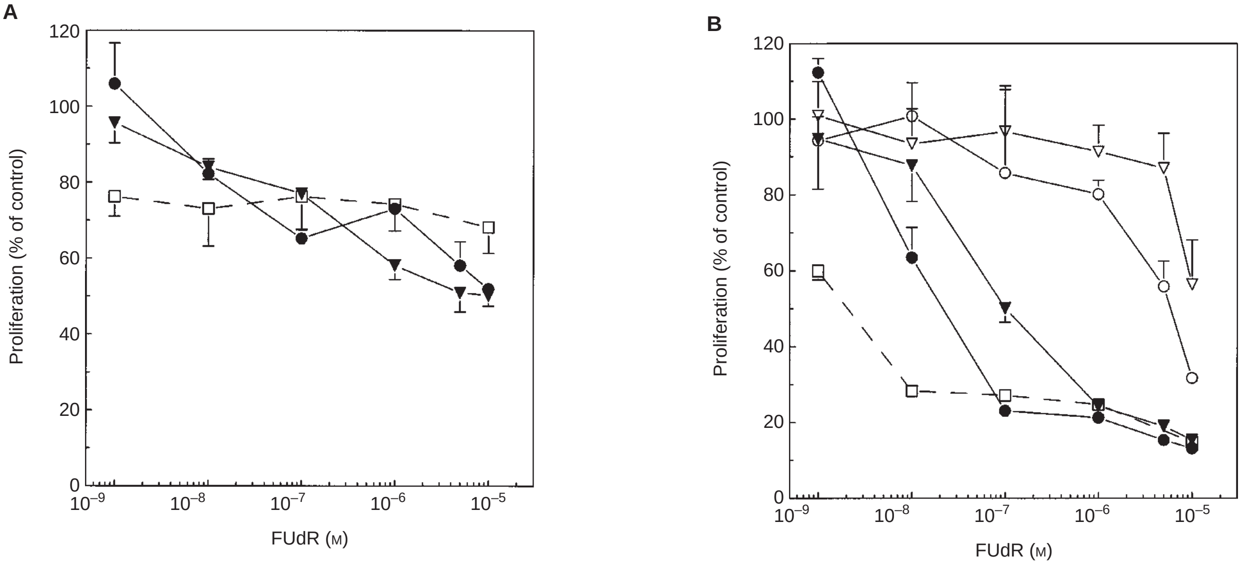

**Figure 3** Effects of different treatment times with 50-nm CC52-PC/Chol-liposomes (40 mol % Chol) without or with FUdR-dP containing $GM_1$ or PEG on the proliferation of CC531 cells. Proliferation is expressed as percentage of the proliferation of untreated cells (average ± s.d.). PEG, no FUdR-dP (∇); $GM_1$, no FUdR-dP (○); PEG, FUdR-dP (▼); GM1, FUdR-dP (●) (all $n = 3$) and free FUdR (□) ($n \geq 6$). (**A**) A 48-h treatment. Only results of FUdR-dP containing liposomes are shown, liposomes without the drug had no effect on the proliferation. (**B**) A 72-h treatment

### FUdR-dP in long circulating immunoliposomes

Figure 3 compares the effect of 48- or 72-h treatment with FUdR-dP in 50-nm CC52-$GM_1$- or PEG-liposomes. Treatment for 24 h did not result in a significant effect on proliferation. Treatment for 48 h (Figure 3A) with FUdR-dP-containing $GM_1$-immunoliposomes or PEG-immunoliposomes caused a slightly but significantly ($P < 0.005$) stronger inhibition of proliferation than free FUdR at concentrations higher than $10^{-6}$ M.

Treatment for 72 h with FUdR-dP in CC52-$GM_1$-liposomes resulted in a slightly stronger inhibition of tumour cell growth than FUdR-dP in CC52-PEG-liposomes (Figure 3B). At concentrations lower than $10^{-7}$ M, free FUdR was the most effective. No effect of cholesterol content in CC52-$GM_1$-liposomes was observed (Figures 2 and 3 and Table 1).

### Treatment (24 h) with FUdR-dP immunoliposomes

In the experiments described thus far, the drug was left in contact with the cells for the full 48-h or 72-h incubation period. In the following experiment we incubated the cells for 24 h with the drug formulation, then removed the medium containing the drug and continued incubation for another 48 h in fresh drug-free medium.

FUdR-dP in CC52-$GM_1$-liposomes at concentrations of $10^{-7}$ M or lower, was more effective than FUdR-dP in CC52-PEG-liposomes and even slightly more effective than the free drug ($P < 0.05$) (Figure 4 and Table 1). FUdR-dP in liposomes without antibody had only minor effects on cell proliferation. Treatment for 24 h with FUdR-dP in CC52-$GM_1$-liposomes at concentrations of $10^{-7}$ M and lower, followed by a drug-free incubation period of 48 h, resulted in antiproliferative effects comparable to those obtained following a continuous treatment for 72 h (Figure 3B and Table 1). A 24-h treatment with FUdR-dP in CC52-PEG-liposomes resulted in lower levels of CC531 growth inhibition than a 72-h continuous treatment.

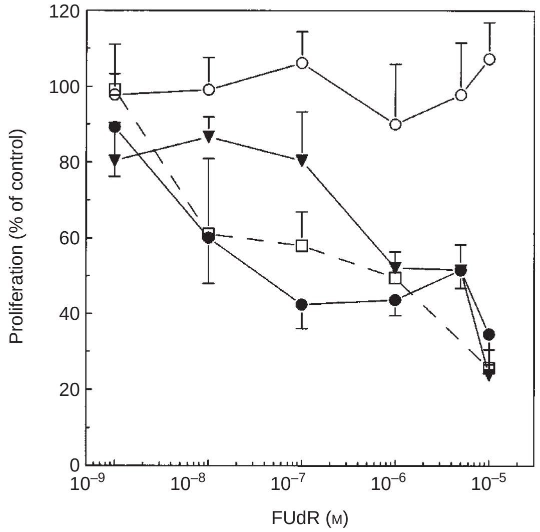

**Figure 4** Antiproliferative effect of liposomal formulations of FUdR-dP; effect of 24-h pre-treatment with 50-nm liposomes followed by 48-h incubation without the drug on the proliferation of CC531 cells. Proliferation is displayed as a percentage of the proliferation of untreated cells (average ± s.d.). FUdR-dP-$GM_1$-liposomes without CC52 (○); CC52-FUdR-dP-PEG-liposomes (▼); CC52-FUdR-dP-$GM_1$-liposomes (●) (all $n = 3$) and free FUdR (□) ($n = 5$)

### Mechanism of interaction of immunoliposomes with CC531 cells and intracellular delivery of FUdR-dP

In the experiment presented in Table 2 association of different radiolabelled liposome formulations with CC531 cells was determined. We compared the CC52-liposomes with liposomes containing irrelevant $IgG_1$ and liposomes without antibody. The latter two showed levels of association of only about 10% of that of tumour cell-specific CC52-liposomes demonstrating the specificity of interaction of the CC52-liposomes with CC531 cells.

 © 1999 Cancer Research Campaign

**Table 2** Association of tumour cell-specific liposomes and control liposomes, with irrelevant murine $IgG_1$ or without antibody coupled, with CC531 colon cancer cells

| Liposome | Association with CC531 cells (nmol lipid per mg cell protein) (± s.d.) |
|---|---|
| CC52-MPB-PEG | 9.67 ± 1.61 |
| $IgG_1$-MPB-PEG | 1.18 ± 0.28 |
| MPB-PEG | 1.00 ± 0.17 |

CC531 cells were incubated for 3 h with $^3$H-COE labelled liposomes (200 nmol ml$^{-1}$). Association is expressed as nmol liposomal lipid and normalized for the amount of cellular protein.

To gain insight in the mechanism of interaction of the immunoliposomes with the tumour cells and the way the drug becomes available inside the cells we incubated the cells with double-labelled immunoliposomes containing both the metabolically degradable ester cholesteryl-[$^{14}$C-]oleate and the non-metabolizable analogue $^3$H-cholesteryloleylether. Following an endocytic uptake process the cholesterylester will be hydrolysed intralysosomally and $^{14}$C-oleate will be released from the cells. Internalized cholesterylether, on the other hand, will remain cell-associated and thus the cellular $^3$H:$^{14}$C ratio is expected to increase when the uptake mechanism involves an endocytic process (Derksen et al, 1988). During a 48-h incubation, the cellular $^3$H:$^{14}$C ratio remains essentially constant irrespective of the presence or absence of serum in the medium, indicating that the cholesterylester is not exposed to a lysosomal environment intracellularly (Figure 5).

Also the fate of the prodrug in the cells was followed by incubating cells for 3 h with CC52-MPB-liposomes containing $^3$H-FUdR-dP followed by a 6-h incubation period without immunoliposomes. Within 3 h, 1.25 ± 0.09 μmol FUdR-dP mg$^{-1}$ cell protein (average of six determinations ± s.e.m.) was associated with the tumour cells. Six hours after ending the incubation with liposomes, the medium contained 0.46 ± 0.05 μmol of FUdR(-dP) mg$^{-1}$ cell protein of which 84 ± 7% was water soluble, representing hydrolysed prodrug. The amount of cell-bound liposomal FUdR-dP was 0.62 ± 0.04 μmol mg$^{-1}$ cell protein, whereas 0.17 ± 0.03 μmol FUdR(-dP) mg$^{-1}$ cell protein was retrieved intracellularly of which 49 ± 11% was water soluble FUdR. These results demonstrate that, in sharp contrast to the immunoliposome-incorporated cholesterylester, immunoliposomal FUdR-dP is rapidly hydrolysed by CC531 cells, resulting in efficient intracellular delivery of active drug.

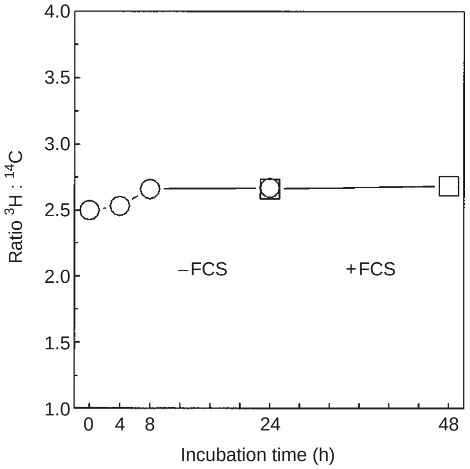

**Figure 5** Lack of immunoliposome degradation by CC531 tumour cells. Ratio $^3$H:$^{14}$C associated with CC531 cells after incubation with CC52-PC/Chol-liposomes double labelled with the non-degradable marker $^3$H-COE and the intracellular degradable marker $^{14}$C-COA, followed in time. Incubations without fetal calf serum (FCS) (○) and with FCS (□) (*n* = 3, standard deviations are within symbols)

### DISCUSSION

The establishment of a tight interaction between liposomes and tumour cells, by means of antibody–antigen interactions, resulted in a major increase in the antiproliferative efficacy and thus, apparently, of the delivery of FUdR-dP to CC531 cells as compared to liposomes without specific antibody. The specificity of the interaction of CC52-liposomes with CC531 cells was confirmed by a tenfold higher level of association of CC52-liposomes compared to liposomes carrying irrelevant IgG or liposomes without antibody.

In order to become effective, liposomal FUdR-dP has to be hydrolysed to the active parent compound FUdR. Van Borssum Waalkes et al (1993) showed that little or no hydrolysis of FUdR-dP occurs in serum. Extensive hydrolysis of FUdR-dP, leading to the virtually complete availability of the active drug in 48 to 72 h, can only occur after uptake of the prodrug and subsequent intracellular hydrolytic removal of the esterified palmitic acid chains (Van Borssum Waalkes et al, 1990). The higher antiproliferative activity towards CC531 colon cancer cells of free FUdR as compared to FUdR-dP in liposomes without antibody (Figure 1) indicates that only a small part of the liposomal prodrug from these liposomes is hydrolysed into FUdR. The increase in antiproliferative activity obtained by coupling a tumour cell-specific antibody to the FUdR-dP-containing liposomes indicates the involvement of a different mechanism in the release of active drug from the immunoliposomes.

Endocytic uptake of the immunoliposomes is unlikely to play a role in the entrance of FUdR-dP into the tumour cells. For example, the similar effectiveness of the 50-nm and the 200-nm immunoliposomes argues against such a mechanism because particles > 120 nm are generally not endocytosed by non-phagocytic cells (Scherphof, 1991). Furthermore, Figure 5 shows that liposomal cholesteryloleate is not hydrolysed by the tumour cells within a timespan of as long as 48 h, this also argues against endocytic uptake of the CC52-liposomes. Nonetheless, the tight interaction of the immunoliposomes with the tumour cells leads to efficient intracellular delivery of FUdR as was confirmed by tracing $^3$H-FUdR-dP and its deacylated metabolites during the interaction of CC52-liposomes with CC531 cells. Considerable amounts of FUdR-dP and hydrolysed prodrug were detected intracellularly, suggesting the involvement of an intracellular hydrolytic process in the release of active FUdR. We postulate a selective transfer of FUdR-dP from the immunoliposomal bilayer to the plasma membrane, followed by tumour cell mediated hydrolysis, yielding effective concentrations of active drug intracellularly.

A transfer of FUdR-dP from the bilayer of immunoliposomes to plasma membranes has been proposed before by us in collaboration with Dr Leaf Huang, University of Pittsburgh, USA. FUdR-dP incorporated in $GM_1$-immunoliposomes targeted to lung endothelium resulted in increased antitumour activity towards lung metastases of EMT6 mouse mammary carcinoma as

*© 1999 Cancer Research Campaign*

compared to non-targeted liposomes. We tentatively ascribed this to a transfer of FUdR-dP from the immunoliposomes to the plasma membrane of the lung endothelial cells (Mori et al, 1995). Since $GM_1$ was previously shown to facilitate selective transfer of liposomal bilayer constituents from liposomes to hepatocytes (Hoekstra et al, 1980), this compound was hypothesized to facilitate transfer of FUdR-dP from liposomes to plasma membranes (Scherphof et al, 1992). However, in our experiments, the presence of $GM_1$ in plain liposomes or in immunoliposomes did not enhance the antiproliferative effect of FUdR-dP towards CC531 cells compared to (immuno)liposomes without $GM_1$ and thus, apparently, $GM_1$ did not augment the transfer of the prodrug to the tumour cells. The FUdR-dP transfer to endothelial cells, as mentioned above, may have been induced by the antibody–antigen mediated interaction between the liposomal bilayer and the plasma membrane, comparable to the transfer-mechanism postulated in this paper. Cellular uptake of a liposome-associated lipophilic derivative of methotrexate has been reported suggesting a similar mechanism of drug uptake independent on internalization of the entire liposome (Kinsky et al, 1987).

The somewhat stronger antitumour activity of 200-nm immunoliposomes as compared to the 50-nm vesicles, which was observed at FUdR-dP concentrations of $10^{-6}$ M and higher, may be explained by the larger number of FUdR-dP molecules incorporated per vesicle in the larger liposomes. Also, more antibody-molecules are coupled per vesicle, allowing a tighter interaction with the tumour cells. For in vivo targeting to tumours, liposomes of 100 nm or smaller are preferable to larger ones because of their longer circulation time (Mayer et al, 1989) and a concomitantly higher chance of extravasation in tumour areas (Papahadjopoulos et al, 1991). The difference in antiproliferative efficacy between PEG-immunoliposomes and $GM_1$-immunoliposomes in our experiments can be ascribed to the three-fold higher amount of antibody coupled to the latter ones. Although immunoliposomes containing $GM_1$ show a higher anti-tumour activity, it may be more advantageous, in vivo, to apply PEG since rat and human serum contain anti-$GM_1$-antibodies which enhance rapid clearance of $GM_1$-liposomes (Liu et al, 1995).

The experiments, in which the anti-tumour effect was measured 48 h after a 24-h treatment with FUdR-dP in CC52-liposomes, illustrate the potential advantage of immunoliposomes over non-targeted liposomal FUdR-dP and free FUdR. This experimental condition is considered to resemble more closely the in vivo situation than the 72-h continuous treatment. By incorporating FUdR-dP in (immuno)liposomes the circulation half-life of the drug is drastically increased as compared to that of free FUdR, which has a circulation half-life of only a few minutes (Ensminger et al, 1978), and besides, immunoliposomes are expected to selectively bind to the tumour cells. Both conditions will result in increased duration of exposure of the tumour to the drug. The presence of antibodies on the liposomal surface is expected to result in increased uptake of immunoliposomes by cells of the mononuclear phagocytic system (Derksen et al, 1998). However, we observed that the presence of PEG on the surface of immunoliposomes attenuates this effect (Scherphof et al, 1997).

For non-targeted, non-long-circulating (conventional) liposomes containing FUdR-dP, increased toxicity compared to free FUdR has been described (Supersaxo et al, 1988; Van Borssum Waalkes et al, 1992). This was attributed to the sustained release of FUdR from Kupffer cells after uptake of the FUdR-dP-liposomes (Van Borssum Waalkes et al, 1992). Mori et al (1995) showed however, that a combination of long-circulating capacity and targeting of the liposomes resulted in improved therapeutic efficacy and less toxicity as compared to conventional liposomes. Our present approach, in which we combine both liposomal parameters to target FUdR-dP to colon cancer cells, shows that CC52-immunoliposomes are able to deliver the active drug to the cancer cells with high efficiency, by a mechanism probably involving the selective transfer of the lipophilic prodrug from the immunoliposomes to the tumour cell membrane. The obvious advantage of this approach over the use of an encapsulated drug is that the uptake of drug proceeds independently of endocytic internalization of the liposomes as such, a property which may be more generally applicable for lipophilic (pro)drugs incorporated in targeted liposomes.

We are currently testing the validity of this formulation with respect to in vivo anti-tumour activity towards liver metastases of colon cancer.

### ACKNOWLEDGEMENT

This work was supported by grant 94-767 from the Dutch Cancer Society.

### REFERENCES

Ahmad I, Longenecker M, Samuel J and Allen TM (1993) Antibody-targeted delivery of doxorubicin entrapped in sterically stabilized liposomes can eradicate lung cancer in mice. *Cancer Res* **53**: 1484–1488

Allen TM (1997) Liposomes: opportunities in drug delivery. *Drugs* **54**: 8–14

Allen TM and Chonn A (1987) Large unilamellar liposomes with low uptake into the reticuloendothelial system. *FEBS Lett* **223**: 42–46

Allen TM, Hansen C, Martin F, Redemann C and Yau Young A (1991) Liposomes containing synthetic lipid derivatives of poly(ethylene glycol) show prolonged circulation half-lives in vivo. *Biochim Biophys Acta* **1066**: 29–36

Beun GD, van Eendenburg DH, Corver WE, van de Velde CJ and Fleuren GJ (1992) T-cell retargeting using bispecific monoclonal antibodies in a rat colon carcinoma model. I. Significant bispecific lysis of syngeneic colon carcinoma CC531 is critically dependent on prolonged preactivation of effector T-lymphocytes by immobilized anti-T-cell receptor antibody. *J Immunother* **11**: 238–248

Beun GD, Gorter A, Nooyen Y, van de Velde CJ and Fleuren GJ (1993) T cell retargeting using bispecific monoclonal antibodies in a rat colon carcinoma model. II. Syngeneic colon carcinoma CC531 is efficiently killed by retargeted cytotoxic T lymphocytes in vitro despite limited lysis in 51Cr release assays. *J Immunol* **150**: 2305–2315

Böttcher CJF, Van Gent CM and Pries C (1961) A rapid and sensitive sub-micro phosphorus determination. *Anal Chim Acta* **24**: 203–204

Danenberg PV (1977) Thymidylate synthetase – a target enzyme in cancer chemotherapy. *Biochim Biophys Acta* **473**: 73–92

Derksen JTP, Morselt HWM and Scherphof GL (1988) Uptake and processing of immunoglobulin-coated liposomes by subpopulations of rat liver macrophages. *Biochim Biophys Acta* **971**: 127–136

Derksen JTP and Scherphof GL (1985) An improved method for the covalent coupling of proteins to liposomes. *Biochim Biophys Acta* **814**: 151–155

Dingemans KP, van den Bergh Weerman MA, Keep RF and Das PK (1994) Developmental stages in experimental liver metastases: relation to invasiveness. *Int J Cancer* **57**: 433–439

Ensminger WD, Rosowsky A, Raso V, Levin DC, Glode M, Come S, Steele G and Frei E (1978) A clinical-pharmacological evaluation of hepatic arterial infusions of 5-fluoro-2′-deoxyuridine and 5-fluorouracil. *Cancer Res* **38**: 3784–3792

Gregoriadis G (1995) Engineering liposomes for drug delivery: progress and problems. *Trends Biotechnol* **13**: 527–537

Hoekstra D, Tomasini R and Scherphof GL (1980) Interactions of phospholipid vesicles with rat hepatocytes in vitro. Influence of vesicle-incorporated glycolipids. *Biochim Biophys Acta* **603**: 336–346

Kamps JAAM, Swart PJ, Morselt HWM, Pauwels R, De Bethune MP, De Clercq E, Meijer DKF and Scherphof GL (1996) Preparation and characterization of

 *© 1999 Cancer Research Campaign*

conjugates of (modified) human serum albumin and liposomes: drug carriers with an intrinsic anti-HIV activity. *Biochim Biophys Acta* **1278**: 183–190

Kemeny NE (1995) Regional chemotherapy of colorectal cancer. *Eur J Cancer* **31A**: 1271–1276

Kemeny N (1996) Colorectal cancer – An undertreated disease. *Anti-Cancer Drugs* **7**: 1–7

Kinsky SC and Loader JE (1987) Circumvention of the methotrexate transport system by methotrexate-phosphatidylethanolamine derivatives: effect of fatty acid chain length. *Biochim Biophys Acta* **921**: 96–103

Liu D, Song YK and Liu F (1995) Antibody dependent, complement mediated liver uptake of liposomes containing $GM_1$. *Pharm Res* **12**: 1775–1780

Lowry OH, Rosebrough NJ, Farr AL and Randall RJ (1951) Protein measurement with the folin phenol reagent. *J Biol Chem* **193**: 265–275

Marinelli A, Dijkstra FR, van Dierendonck JH, Kuppen PJ, Cornelisse CJ and van de Velde CJ (1991) Effectiveness of isolated liver perfusion with mitomycin C in the treatment of liver tumours of rat colorectal cancer. *Br J Cancer* **64**: 74–78

Marquet RL, Westbroek DL and Jeekel J (1984) Interferon treatment of a transplantable rat colon adenocarcinoma: importance of tumor site. *Int J Cancer* **33**: 689–692

Martin FJ and Papahadjopoulos D (1982) Irreversible coupling of immunoglobulin fragments to preformed vesicles. An improved method for liposome targeting. *J Biol Chem* **257**: 286–288

Maruyama K, Kennel SJ and Huang L (1990) Lipid composition is important for highly efficient target binding and retention of immunoliposomes. *Proc Natl Acad Sci USA* **87**: 5744–5748

Mayer LD, Tai LC, Ko DS, Masin D, Ginsberg RS, Cullis PR and Bally MB (1989) Influence of vesicle size, lipid composition, and drug-to-lipid ratio on the biological activity of liposomal doxorubicin in mice. *Cancer Res* **49**: 5922–5930

Meropol NJ, Creaven PJ and Petrelli NJ (1995) Metastatic colorectal cancer: advances in biochemical modulation and new drug development. *Semin Oncol* **22**: 509–524

Mori A, Kennel SJ, Van Borssum Waalkes M, Scherphof GL and Huang L (1995) Characterization of organ-specific immunoliposomes for delivery of 3′,5′-O-dipalmitoyl-5-fluoro-2′-deoxyuridine in a mouse lung-metastasis model. *Cancer Chemother Pharmacol* **35**: 447–456

Myers CE (1981) The pharmacology of the fluoropyrimidines. *Pharmacol Rev* **33**: 1–15

Nishizawa Y and Casida JE (1965) 3′,5′-diesters of 5-fluoro-2′-deoxyuridine: synthesis and biological activity. *Biochem Pharmacol* **14**: 1605–1619

Papahadjopoulos D, Allen TM, Gabizon A, Mayhew E, Matthay K, Huang SK, Lee KD, Woodle MC, Lasic DD, Redemann C and Martin FJ (1991) Sterically stabilized liposomes: improvements in pharmacokinetics and antitumor therapeutic efficacy. *Proc Natl Acad Sci USA* **88**: 11460–11464

Petterson GL (1977) A simplification of the protein assay method of Lowry et al. which is more generally applicable. *Anal Biochem* **83**: 346–356

Scherphof GL (1991) In vivo behaviour of liposomes: Interactions with the mononuclear phagocyte system and implications for drug targeting. In: *Handbook of Experimental Pharmacology*, Juliano RL (ed), pp. 285–327. Springer Verlag: Berlin

Scherphof GL, Maruyama K, Van Borssum Waalkes M, Hoekstra D, Damen J, Kennel SJ and Huang L (1992) Lipid flow phenomena between liposomes, lipoproteins and cell membranes: applications in drug delivery. In: *Liposome Dermatics*, Braun-Falco O, Korting HC and Maibach HI (eds), pp. 11–19. Springer Verlag: Berlin

Scherphof GL, Kamps JAAM and Koning GA (1997) In vivo targeting of surface-modified liposomes to metastatically growing colon carcinoma cells and sinusoidal endothelial cells in the rat liver. *J Liposome Res* **7**: 419–432

Schwendener RA, Supersaxo A, Rubas W, Weder HG, Hartmann HR, Schott H, Ziegler A and Hengartner H (1985) 5′-O-Palmitoyl- and 3′,5′-O-dipalmitoyl-5-fluoro-2′-deoxyuridine – novel lipophilic analogues of 5′-fluoro-2′-deoxyuridine: synthesis, incorporation into liposomes and preliminary biological results. *Biochem Biophys Res Commun* **126**: 660–666

Storm G and Crommelin DJA (1997) Colloidal systems for tumor targeting. *Hybridoma* **16**: 119–125

Supersaxo A, Rubas W, Hartmann HR, Schott H, Hengartner H and Schwendener RA (1988) The antitumour effect of lipophilic derivatives of 5-fluoro-2′-deoxyuridine incorporated into liposomes. *J Microencapsul* **5**: 1–11

Thomas C, Nijenhuis AM, Timens W, Kuppen PJ, Daemen T and Scherphof GL (1993) Liver metastasis model of colon cancer in the rat: immunohistochemical characterization. *Invasion Metast* **13**: 102–112

Vaage J, Donovan D, Uster P and Working P (1997) Tumour uptake of doxorubicin in polyethylene glycol-coated liposomes and therapeutic effect against a xenografted human pancreatic carcinoma. *Br J Cancer* **75**: 482–486

Vahrmeijer AL, van Dierendonck JH and van de Velde CJ (1995) Treatment of colorectal cancer metastases confined to the liver. *Eur J Cancer* **31A**: 1238–1242

Van Borssum Waalkes M and Scherphof GL (1990) Liposome-incorporated 3′,5′-O-dipalmitoyl-5-fluoro-2′-deoxyuridine as a slow-release antitumor drug depot in rat liver macrophages. *Selective Cancer Ther* **6**: 15–22

Van Borssum Waalkes M, Fichtner I, Dontje B, Lemm M, Becker M, Arndt D and Scherphof GL (1992) In vivo distribution and antitumour activity of liposomal 3′,5′-O-dipalmitoyl-5-fluoro-2′-deoxyuridine. *J Microencapsul* **9**: 335–346

Van Borssum Waalkes M, Van Galen M, Morselt HWM, Sternberg B and Scherphof GL (1993) In-vitro stability and cytostatic activity of liposomal formulations of 5-fluoro-2′-deoxyuridine and its diacylated derivatives. *Biochim Biophys Acta* **1148**: 161–172

© 1999 Cancer Research Campaign
